# New Onset of Autoimmune Diseases Following COVID-19 Diagnosis

**DOI:** 10.3390/cells10123592

**Published:** 2021-12-20

**Authors:** Abraham Edgar Gracia-Ramos, Eduardo Martin-Nares, Gabriela Hernández-Molina

**Affiliations:** 1 Departamento de Medicina Interna, Hospital General, Centro Médico Nacional “La Raza”, Instituto Mexicano del Seguro Social, Mexico City 02990, Mexico; 2 Sección de Estudios de Posgrado e Investigación, Escuela Superior de Medicina, Instituto Politécnico Nacional, Mexico City 11340, Mexico; 3 Department of Immunology and Rheumatology, Instituto Nacional de Ciencias Médicas y Nutrición Salvador Zubirán, Mexico City 14080, Mexico; eduardomartinnares@gmail.com (E.M.-N.); gabyhm@yahoo.com (G.H.-M.)

**Keywords:** COVID-19, SARS-CoV-2, autoimmunity, rheumatic diseases

## Abstract

There is growing evidence that coronavirus disease 2019 (COVID-19) can lead to a dysregulation of the immune system with the development of autoimmune phenomena. The consequence of this immune dysregulation ranges from the production of autoantibodies to the onset of rheumatic autoimmune disease. In this context, we conducted a systematic review to analyze the current data regarding the new-onset systemic and rheumatic autoimmune diseases in COVID-19 patients. A literature search in PubMed and Scopus databases from December 2019 to September 2021 identified 99 patients that fulfilled the specific diagnostic/classification criteria and/or nomenclature for each rheumatic autoimmune disease. The main diseases reported were vasculitis and arthritis. Idiopathic inflammatory myopathies, systemic lupus erythematosus, and sarcoidosis were also reported in a limited number of patients, as well as isolated cases of systemic sclerosis and adult-onset Still’s disease. These findings highlight the potential spectrum of systemic and rheumatic autoimmune diseases that could be precipitated by SARS-CoV-2 infection. Complementary studies are needed to discern the link between the SARS-CoV-2 and new onset-rheumatic diseases so that this knowledge can be used in early diagnosis and the most suitable management.

## 1. Introduction

Historically, viral infections have had a complex relationship with a variety of autoimmune diseases such as systemic lupus erythematosus (SLE), rheumatoid arthritis (RA), Sjögren’s syndrome (SS), systemic vasculitis, celiac disease, and multiple sclerosis [1,2]. Examples of viruses that play a role in triggering autoimmune disease include hepatitis C virus, hepatitis B virus, Chikungunya virus, parvovirus B19, herpes viruses, and others [2,3]. The mechanisms underlying the association between viruses and autoimmunity remain poorly understood. Traditionally, cross-reactive T-cell recognition, known as molecular mimicry, as well as bystander T-cell activation, culminating in epitope spreading, were the predominant mechanisms by which infection can lead to a T-cell-mediated autoimmune response. However, other hypotheses including virus-induced decoy of the immune system also warrant discussion regarding their potential for triggering autoimmunity [4,5].

There is growing evidence that infection with severe acute respiratory syndrome coronavirus 2 (SARS-CoV-2) is associated with the development of autoimmunity phenomena. Several studies have reported the presence of autoantibodies in patients with coronavirus disease 2019 (COVID-19) in different frequencies: antinuclear antibodies (ANA) in 35.6%, anti-Ro/SSA in 25%, rheumatoid factor in 19%, lupus anticoagulant in 11% and antibodies against interferon (IFN)-I in 10% [6,7,8,9]. Moreover, 28 human proteins with homologous regions to SARS-CoV-2 peptides were identified that could potentially function as autoantigens in COVID-19 patients displaying autoimmune conditions [10]. Autoantibodies against those proteins were detected in typical autoimmune conditions [10]. Other hypothetical mechanisms include bystander activation triggered by a hyper-inflammatory state (often referred to as “cytokine storm” or “cytokine release syndrome”), viral persistence (polyclonal activation due to the constant presence of viral antigens driving immune-mediated injury) and the formation of neutrophil extracellular traps [1,11,12].

The clinical spectrum of autoimmune-related manifestations in COVID-19 patients ranges from organ-specific (e.g., cutaneous vasculitis, immune thrombocytopenic purpura, transverse myelitis, Guillain–Barré syndrome) to systemic autoimmune and inflammatory conditions (e.g., systemic vasculitis, multisystem inflammatory syndrome (MIS)), hemophagocytic lymphohistiocytosis (HLH), SLE [12,13]. The objective of this systematic review is therefore to discuss and show current data regarding new-onset rheumatic autoimmune diseases following COVID-19 in adult patients, considering only those cases that fulfilled the specific diagnostic/classification criteria and/or nomenclature for each disease.

## 2. Search Strategy

The search process was carried out following the PRISMA guideline [14]. PubMed and Scopus databases were searched for relevant publications from December 2019 (when SARS-CoV-2 was first reported) to 22 September 2021. Publications were searched specifically using the terms: “severe acute respiratory syndrome coronavirus 2” “Coronavirus”, “COVID-19”, “Arthritis”, “Vasculitis”, “Systemic Vasculitis”, “Myositis”, “Polymyositis”, “Rhabdomyolysis”, “Lupus Erythematosus, Systemic”, “Antiphospholipid Syndrome”, “Still’s Disease, Adult-Onset”, “Sarcoidosis”, “Scleroderma, Systemic”, “Scleroderma, Diffuse”, “Sjogren’s Syndrome”. In addition, the references and related citations for the resulting articles were also reviewed for inclusion. Publications without language restrictions were eligible for inclusion. Cohort studies, case reports or case series of patients with acute or previous SARS-CoV-2 infection were included if the patients fulfilled the specific diagnostic/classification criteria and/or nomenclature for each rheumatic autoimmune disease: 2010 RA classification criteria [15], the Fourth International workshop on reactive arthritis (ReA) [16], 2012 Chapel Hill Consensus Conference nomenclature (2012 CHCC) in single-organ vasculitides (SOV) or systemic vasculitides [17], 1990 American College of Rheumatology (ACR) criteria for giant cell arteritis (GCA) [18]; 1990 ACR criteria for polyarteritis nodosa (PAN) [19]; the European Medicines Agency Consensus algorithm for ANCA-associated vasculitides (AAV) and PAN [20]; the Centers for Disease Control and Prevention (CDC) case definition and/or the American Heart Association criteria for complete and incomplete Kawasaki disease [21,22], the 2012 CHCC for GCA, AAV, PAN, IgA vasculitis, anti-glomerular basement membrane (anti-GBM) disease, primary central nervous system vasculitis, cutaneous vasculitis and isolated aortitis [17], Kanegaye criteria for Kawasaki disease shock syndrome (KDSS) [23], the CDC case definition for Multysystem Inflammatory Syndrome in adults (MIS-A) and children (MIS-C) [24,25], the Bohan and Peter criteria for dermatomyositis (DM) and polymyositis (PM) [26,27], 2017 European League Against Rheumatism (EULAR)/ACR classification criteria for adult and juvenile idiopathic inflammatory myopathies and their major subgroups [28], 2019 EULAR/ACR classification criteria for systemic lupus erythematosus [29], 2016 ACR /EULAR SS classification criteria [30], Yamaguchi criteria for adult-onset Still’s disease (AOSD) [31], ASAS criteria for axial and peripheral spondyloarthritis (SpA) [32], Joint Statement of the American Thoracic Society (ATS), the European Respiratory Society (ERS) and the World Association of Sarcoidosis and Other Granulomatous Disorders (WASOG), sarcoidosis criteria [33], and the International Consensus Statement on an update of the classification criteria for definite antiphospholipid syndrome (APS) [34].

Publications were excluded if they did not fulfill the above criteria or if they included patients under 18 years of age. The following data were extracted from the studies: age, sex, country of publication, COVID-19 diagnosis method, COVID-19 severity, days from SARS-CoV-2 infection to first symptom attributable to rheumatic autoimmune disease, COVID-19 outcome, rheumatic autoimmune disease organ involvement, autoantibodies, other diagnostic tests (serology, imaging, synovial fluid analysis, cultures, etc.), and rheumatic autoimmune diagnosis and outcome.

## 3. Results

**Figure 1:**
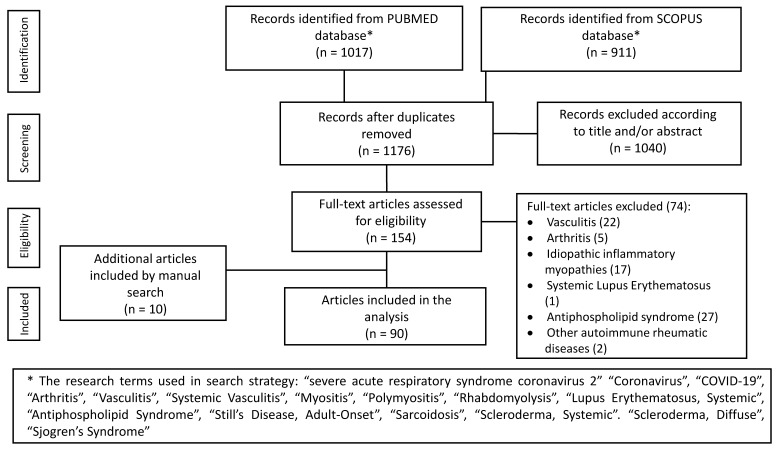
Flow chart of study selection.

**Figure 2:**
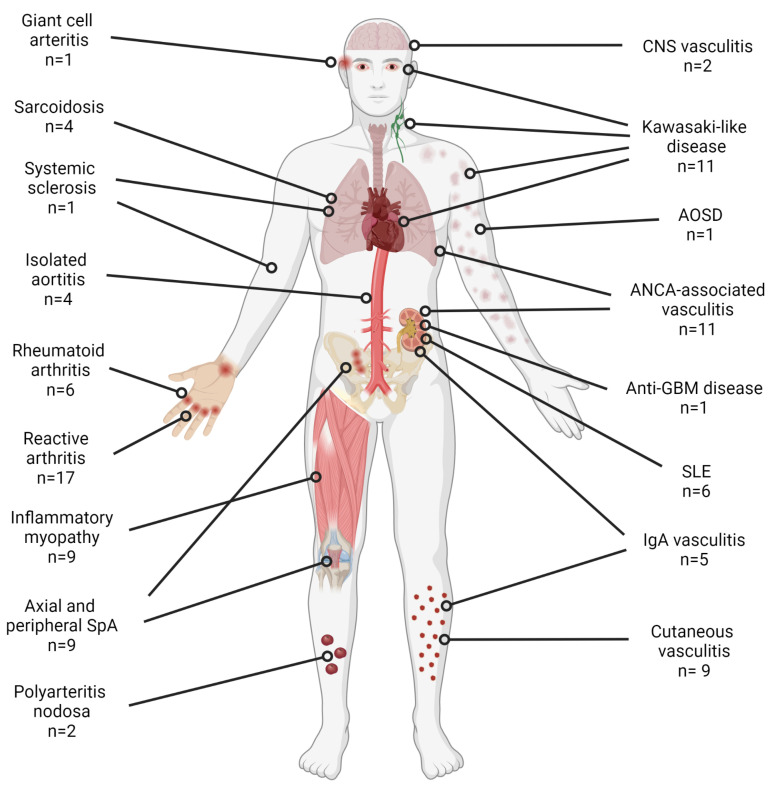
Number of cases and type of new-onset rheumatic autoimmune diseases reported during or after COVID-19. Created with BioRender.com.

The electronic search yielded a total of 1928 publications. After removing duplicate citations, titles and abstracts of 1176 articles were evaluated, and 154 relevant articles were reviewed in detail. Ten articles were identified from the citations of analyzed articles. Finally, 90 reports (99 cases) were included (Table 1, Figure 1 and Figure 2).

### 3.1. Vasculitis

Sixty-five articles reporting 70 patients with vasculitic manifestations during or after COVID-19 infection were retrieved. We excluded 24 patients: 10 did not have objective evidence of vasculitis, 7 did not fulfill established criteria, 3 where SARS-CoV-2 was the direct causative agent, 2 with insufficient information, 1 with no proven COVID-19 and 1 duplicated case. Forty-six cases from 43 articles fulfilled criteria and/or nomenclature for immune-mediated vasculitides and were included [35,36,37,38,39,40,41,42,43,44,45,46,47,48,49,50,51,52,53,54,55,56,57,58,59,60,61,62,63,64,65,66,67,68,69,70,71,72,73,74,75,76,77] (Supplementary file).

Nineteen cases were reports from Europe, 17 from North America and 10 from Asia. The mean age of the patients was 46.9 ± 17.6 years and 28 (60.8%) were men. The diagnosis of COVID-19 was established by real-time polymerase chain reaction (RT-PCR) in 27, anti-SARS-CoV-2 IgG and/or IgM antibodies in 9, both RT-PCR and anti-SARS-CoV-2 antibodies in 6, CT findings in 2, in 1 case by clinical findings and epidemiological background, and in 1 patient this information was not available. The severity of COVID-19 was classified as asymptomatic in 4 (8.7%), mild in 15 (32.6%), moderate in 16 (34.8%), severe in 5 (10.8%) and critical in 3 (6.5%); no information was available in 3 cases. In 21 (45.6%) cases, vasculitis presented concomitant and in 21 (45.7%) at a mean of 28.7 ± 15.1 days after SARS-CoV-2 infection; no information was available in 4 cases. Regarding the SARS-CoV-2 infection outcome, only two patients died, no information was reported in three patients, and infection had resolved in the rest of them.

Organs involved by the vasculitic process were the skin in 28 (60.8%), the kidney in 14 (30.4%), the eyes in 10 (21.7%), lymph nodes and heart in 9 (19.6%) each, the lungs in 7 (15.2%), the joints in 6 (13%), the aorta in 5 (10.8%), the gastrointestinal system in 4 (8.7%), the liver in 3 (6.6%), the ENT region and the brain in 2 (4.3%) each, and the spleen, temporal arteries and iliac arteries in 1 (2.1%) each. In 31 (67.4%) patients a biopsy of the involved organs was performed and in 27 (58.7%) there was histopathological proof of vasculitis. Reports consisted of 15 cases of SOV and 31 of systemic vasculitides. Twenty-eight (60.8%) cases were considered small-vessel, 13 medium-vessel (28.3%) and 5 large-vessel vasculitides (10.8%) (Table 1). The specific diagnoses of SOV were cutaneous vasculitis in 9 (all had a biopsy available), isolated aortitis in 4, central nervous system (CNS) vasculitis in 2. The diagnoses of systemic vasculitides were Kawasaki disease in 11, AAV in 11, IgA vasculitis in 5, PAN in 2, and giant cell arteritis (GCA) and anti-GBM disease in 1 each.

Concerning adult-onset Kawasaki disease reports, 7 fulfilled criteria for complete and 4 for incomplete Kawasaki disease; while only 7 patients fulfilled criteria for KDSS, all patients fulfilled the case definition for MIS, 10 met the MIS-A and one met the MIS-C definition. Regarding AAV clinicopathological phenotype 7 were microscopic polyangiitis (MPA), 2 were granulomatosis with polyangiitis, and 2 were renal-limited vasculitis. Finally, concerning PAN, 1 case was systemic and 1 was cutaneous PAN.

Vasculitis treatment consisted of systemic glucocorticoids in 27 cases, intravenous immunoglobulin in 10, rituximab in 6, cyclophosphamide and plasmapheresis in 4 each, tocilizumab in 3, mycophenolate mofetil and topical glucocorticoids in 2 each, and anakinra in 1. Other treatments were aspirin in 4 cases, anticoagulation in 3 cases, NSAIDs in 2 cases, renal replacement therapy in 2 cases and antihistamines in 2 cases. In 4 reports the treatment prescribed for vasculitis was not reported. Vasculitis resolved in 28 patients, improved in 9 and 4 patients died, of which only 1 was attributed to the vasculitic process.

### 3.2. Arthritis

Thirty-three papers reporting 43 patients were retrieved for detailed analysis. Then we excluded 11 patients: 4 with a gout attack, 1 with established RA, 1 with isolated dactylitis, 1 patient who had already been investigated for arthritis before COVID-19 and 4 patients with back pain and sacroiliitis by MRI but with an age at onset > 45 years. Therefore, our review related to arthritis was based on 28 studies [78,79,80,81,82,83,84,85,86,87,88,89,90,91,92,93,94,95,96,97,98,99,100,101,102,103,104,105] (Supplementary file). All included studies were published as full-text journal articles. A total of 16 studies were from Europe, 9 from Asia and 3 from America. The mean age of the patients was 49.5 ± 15.1, and 18 of them (56.2%) were men. The diagnosis of SARS-CoV-2 infection was established by a positive nasopharyngeal swab in 25 patients, and in 17 of these patients, the diagnostic method was RT-PCR. In 4 patients, the diagnosis was established on the basis of previous symptoms, epidemiological background, and anti-SARS-CoV-2 serology; and in 4 patients, no information was available. The severity of SARS-CoV-2 infection was classified as mild in 65.5% of the cases, moderate in 9.3%, severe in 21.8% and critical in 3.1% of the cases. Regarding the SAR-CoV-2 infection outcome, only 1 patient had died, no information was available in 4 patients, and the infection had resolved in the rest.

All studies except 2, reported the number of days from COVID-19 infection to the onset of arthritis, with a mean of 20.2 ± 11.5 days. Three patients debuted with axial involvement and 29 with peripheral arthritis (9 monoarticular and 20 polyarticular). In addition, 3 patients had concomitant enthesitis. There were 2 patients with psoriatic lesions and 1 patient with balanitis.

HLA-B27 antigen was tested in 8 patients and was positive in 2 patients (25%); whereas rheumatoid factor was tested in 18 patients and was positive in 2. The most common imaging modalities were MRI (sacroiliac joint or peripheral joint) in 6 patients, and ultrasound in 5 patients. Synovial fluid analysis was performed in 10 patients, which ruled out infection and crystals. In 1 patient, the anti-SARS-CoV2 IgG antibody in the synovial fluid was positive, and in 2 patients the test for SARS-CoV-2 RT-PCR was negative.

As for rheumatologic diagnosis, 6 patients had RA, 3 had axial SpA and 6 had peripheral SpA. The remaining 17 patients did not fulfill the ASAS classification for SpA, as they had isolated arthritis in the context of post-COVID-19.

Intraarticular steroids were used in 4 patients, intramuscular in 1 patient and oral in 7 patients. Most patients (n = 17) received NSAIDS (alone or in combination with steroids). Methotrexate or sulfasalazine were used in only 3 patients, certolizumab in 1 patient, and baricitinib in 1 patient. Only 1 patient received no treatment, and treatment was not specified in 5 cases.

In most patients, the arthritis resolved without treatment, with the exception of RA patients who were in remission or showed improvement but were still under treatment.

### 3.3. Idiopathic Inflammatory Myopathies

Forty-six patients from 25 articles concerning myositis and SARS-CoV-2 infection were retrieved for detailed analysis. Were excluded 37 patients: 35 did not fulfill establishing criteria and 2 patients had a previous diagnosis of dermatomyositis. Therefore, 9 cases from 8 articles were included in this review. Six cases fulfilled the 2017 EULAR/ACR classification criteria for adult and juvenile IIM and 3 patients were classified as possible polymyositis according to the Bohan and Peter criteria for DM and PM [106,107,108,109,110,111,112,113] (Supplementary file). Four cases were reports from Europe, 3 from America, and 2 from Asia. The mean age of the cases was 55.6 (range 38–77) years and 5 cases were women; in 1 case, age and sex were not reported. The diagnosis of COVID-19 was made by RT-PCR test in 8 patients and in 1 patient by serologic test. The time from SARS-CoV-2 infection to IIM onset varied from 10 days to 3 months.

The severity of SARS-CoV-2 infection was reported as severe in 4 cases, critical, moderate, and mild in 1 case each; in two cases the severity of COVID-19 was not reported. In 3 cases, myositis symptoms began after COVID-19 resolution.

All patients presented proximal muscle weakness in the upper and lower extremities; myalgia occurred in 3 patients, dysphagia in 2 patients, typical rash in 2 patients and respiratory muscle weakness in one patient. One patient had cardiac involvement. Muscle biomarkers were measured in 10 patients, with an increase in creatin kinase level in 9 cases (range 150–32230 U/L).

Immunological tests showed positive ANA in 3 cases, positive anti-small ubiquitin-like modifier-1 activating enzyme (anti-SAE1) antibody in 2 cases, positive anti-Ku antibodies in 2 cases and positive anti-MI 2b, positive anti-Ro/SSA antibodies, positive anti-Smith antibody and positive anti-melanoma differentiation-associated protein 5 (MDA-5) in 1 case each; autoantibodies were negative in 5 cases. Electromyography was performed in 3 patients, 2 of them displayed and myopathic pattern. A muscle biopsy was performed in 2 cases and showed acute necrotizing myositis (n = 1), and perivascular inflammatory infiltration with endomysial extension (n = 1). The final diagnosis was polymyositis in 3 cases, dermatomyositis in 2 cases, immune-mediated necrotizing myopathy in 2 cases, anti-SAE1+ IIM in one case and anti-Mi2b+ IIM in 1 case.

Concerning treatment, corticosteroids were used in 8 cases (in 4 cases as monotherapy). Other immunosuppressors used were methotrexate, intravenous immunoglobulin, cyclophosphamide, azathioprine, and mycophenolate mofetil. In 1 case, treatment was not specified. A favorable outcome was reported in 7 patients, and only 1 patient dead (due to SARS-CoV-2 infection); in 1 patient, the clinical course was not reported.

### 3.4. Systemic Lupus Erythematosus

We evaluated 7 articles that describe 7 patients with SLE and COVID-19. One case did not fulfill the diagnostic criteria and therefore, we finally included only 6 cases [114,115,116,117,118,119] (Supplementary file). Patients’ age ranged from 18 to 85 years. Four patients were women. Reports were from North America (2), Africa (2), Europe (1) and Asia (1). The diagnosis of COVID-19 was confirmed by RT-PCR in 4 cases and using serological methods in 2 cases. In 4 patients, the manifestations of SLE began during the acute phase of COVID-19 (with moderate illness in 2 cases, and critical illness in 2 cases).

Organ-specific manifestations due to SLE were renal (proteinuria (5), acute renal injury (3)) and hematological (thrombocytopenia (6), leukopenia (1)) in all cases, serosal involvement in 4 cases, (pleural effusion (3), pericardial effusion (2), ascites (1)) and cutaneous in 3 cases. Immunological studies showed positive ANA (6 cases), hypocomplementemia (6 cases), positive anti-dsDNA antibodies (5 cases), positive antiphospholipid antibodies (3 cases), positive anti-Ro/SSA (2 cases), positive anti-La/SSB (2 cases), positive anti-histone (1 case), positive anti-RNP (1 case), and positive anti-β2-glycoprotein I antibodies (1 case). One case was simultaneously diagnosed with HLH. The patients with positive anti-β2-glycoprotein I antibodies did not fulfill the criteria for antiphospholipid syndrome. Concerning management for SLE activity, all patients underwent corticosteroids (either methylprednisolone or dexamethasone or prednisone) and hydroxychloroquine/chloroquine was added in 3 patients; other treatments included rituximab, cyclophosphamide, plasmapheresis and intravenous immunoglobulin. One patient underwent splenectomy. Three patients required 3 or more immunosuppressors for the management of SLE activity. In relation to the clinical course, death was reported in 2 cases.

### 3.5. Other Rheumatic Autoimmune Diseases

Regarding this group, we evaluated 6 studies that described 8 patients. One patient was excluded as they only had anti-Ro/SSA positivity in the context of fever, and another one only had erythema nodosum. Thus, we finally included 1 patient with systemic sclerosis, 1 with AOSD and 4 with sarcoidosis [120,121,122,123,124] (Supplementary file).

The patient with systemic sclerosis had skin, gastrointestinal, muscular and incipient lung involvement. He also had positive anti-PM/Scl 75/100 antibodies; disease onset was 74 days post-COVID-19 infection. He did not receive immunosuppressors or prednisone, but he remained under surveillance [120]. On the other hand, the AOSD patient was extensively evaluated because of fever, sore throat, rash and arthralgias that presented 44 days post-COVID-19. After ruling out infections and malignancy, the diagnosis was established. This patient received high doses of steroids and anakinra with significant improvement of clinical symptoms. In both cases, COVID-19 severity was mild [121].

Finally, the mean age of the patients with sarcoidosis was 52.5 ± 16.4 years, all were females, and the mean onset of days from COVID-19 to sarcoidosis was 17 ± 9.5. Three patients had cutaneous involvement and two lymphadenopathies. One of the patients also had Löfgren syndrome; all the cases resolved, one patient with the use of topical steroids, another one without treatment, and in the rest, no information was available [122,123,124].

### 3.6. Antiphospholipid Syndrome

Regarding antiphospholipid syndrome during or after COVID-19, 27 articles (26 cohort studies and 1 case report) were retrieved. However, antiphospholipid syndrome criteria were not met by any of the patients, due to insufficient information and/or lack of serial antiphospholipid antibodies testing.

## 4. Discussion

The clinical spectrum of autoimmune-related manifestations in patients with COVID-19 varies over a wide range from organ-specific to systemic disorders. We found that the most common new-onset rheumatic autoimmune diseases during or after SARS-CoV-2 infection are vasculitis and arthritis. IIM, SLE, and sarcoidosis were also described in a limited number of cases, as well as isolated cases of systemic sclerosis and AOSD.

According to the 2012 CHCC, vasculitides can be broadly classified into infectious and noninfectious vasculitides [17]. Direct invasion of microorganisms such as bacteria or viruses is a common cause of vasculitis. Examples include syphilitic aortitis, rickettsial vasculitis and tuberculous cerebral vasculitis. However, vasculitis arising during or after an infectious process without direct invasion of the pathogen to the vessel wall is well-recognized. In such cases, an inflammatory or autoimmune reaction triggered by the pathogen causes a vasculitic syndrome. Examples include hepatitis C virus-associated cryoglobulinemic vasculitis or hepatitis B virus-associated PAN [17].

Since the early months of the COVID-19 pandemic, vasculitis-like manifestations and full-blown vasculitic syndromes were reported mainly in children and adolescents. Post-COVID-19 acral skin lesions or chilblains, the so-called COVID-19 toes were confirmed in some reports were a result of endothelial damage induced by the virus [125]. Cases of cerebral and gastrointestinal vasculitis were also reported. However, in most of those reports, it is believed that the underlying mechanism is a vasculitis mimic, such as disseminated intravascular coagulation or systemic thrombosis rather than a primary vasculitis [126]. However, a syndrome resembling the clinical characteristics and laboratory findings of Kawasaki disease, designated MIS-C, was described as a condition not caused by ongoing viral infection [127,128,129]. Not surprisingly, further reports of different types of vasculitides emerged and it became evident that COVID-19 was capable of inducing vasculitis without directly damaging the vessel wall, as demonstrated by the absence of SARS-CoV-2 in the skin biopsies [38]. Type 3 hypersensitivity is thought to be the mechanism by which COVID-19 may elicit a vasculitic syndrome, at least in the immune complex small vessel vasculitides such as limited-cutaneous and IgA vasculitis [17,130]. This type of inflammatory response appears days to weeks after the initial antigenic challenge and is due to the failure of the innate immune system to clear from the circulation immune complexes, which later precipitate inside tissues and elicit an inflammatory response mediated by complement activation [130].

In our systematic review, we found cases of small, medium and large vessel vasculitides. The large vessel group was the least frequent. One of the most common vasculitides encountered in our systematic review was Kawasaki disease. The occurrence of Kawasaki or Kawasaki-like disease post-COVID-19 is not unexpected, as Kawasaki disease was reported concurrent or following several viral infections such as rhinovirus, enterovirus, adenovirus, other coronaviruses, and dengue virus [131,132,133,134].

It remains a matter of debate whether the Kawasaki-like disease associated with COVID-19 is different than classic Kawasaki disease. In the early months of the pandemic, Verdoni et al., compared the clinical characteristics and outcomes of 10 Kawasaki-like disease pediatric patients who presented after the beginning of the SARS-CoV-2 pandemic versus 19 patients with pre-pandemic Kawasaki disease and found that the former group was older, had a higher rate of cardiac involvement and more frequently evolved to KDSS and macrophage activation syndrome [127]. In the same manner, Pouletty et al. found that Kawasaki-like disease patients with evidence of past SARS-CoV-2 infection presented at an older age, with lower platelet counts, higher incidence of myocarditis and pericarditis and more resistance to first IVIg treatment compared to a historical cohort of 220 classical Kawasaki disease patients [128]. Other studies have also reported higher frequencies of initial gastrointestinal and neurological involvement and higher levels of ferritin and CRP in Kawasaki-like disease associated with SARS-CoV-2 infection [135,136,137]. The aforementioned studies reported only pediatric patients and whether those findings may be extrapolated to the adult population is not known. However, most of the adult patients fulfilling the criteria for Kawasaki disease in this systematic review presented as the pediatric group, with cardiac involvement in the form of myocarditis and half of them with features of KDSS.

The current evidence suggests that Kawasaki-like disease patients may be a phenotype of MIS, as all these patients also fulfilled the CDC’s case definition for MIS-A or MIS-C. On the contrary, in a recent systematic review including 221 MIS-A patients reported in the literature and at the CDC’s MIS-C surveillance system, only 10 out of 221 patients with MIS-A fulfilled criteria for Kawasaki disease [24,25,57]. There is evidence from the pediatric population that MIS-C and classic Kawasaki disease patients present with different T cell subsets, cytokines, chemokines and biomarkers associated with arterial damage [137,138]. Consiglio et al. found lower naïve CD4+ T cells, T follicular helper cells and higher central and effector memory subpopulations and IL-17A levels in MIS-C compared to pre-COVID-19 Kawasaki disease patients, suggesting a different immunopathology between both conditions [137]. Another study by Rodriguez-Smith et al., found that MIS-C patients had significantly higher levels of serum CXCL9, an IFNγ-induced chemokine, compared to pre-COVID-19 Kawasaki disease patients [138].

The aforementioned differences in clinical presentation and immunopathology suggest that Kawasaki disease following SARS-CoV-2 infection may be a different entity, part of the spectrum of MIS. Thus, probably the term Kawasaki-like disease is more appropriate. However, even if they are different entities, it is recommended that patients that fulfilled KD criteria must be treated in the same manner, with IVIg as first-line treatment [139].

On the other hand, all AAV clinicopathological phenotypes, except for eosinophilic granulomatosis with polyangiitis, were reported. Of note, most of the cases had kidney and/or lung involvement. Different mechanisms of kidney injury may ensue in patients with COVID-19, and acute tubular injury is the most common kidney pathology finding [140]. However, if a patient with COVID-19 and kidney injury presents with abnormalities in the urinalysis suggestive of glomerular injuries such as red blood cell casts or dysmorphic erythrocytes, or if kidney injury presents after resolution of the infection, AAV should be considered in the differential diagnosis and a kidney biopsy should be performed [53]. In the same vein, COVID-19 may rarely present with diffuse alveolar hemorrhage and in such cases, AAV or anti-GMB disease should be ruled out [141,142].

The presence of ANCA was detected in patients with SARS-CoV-2 infection without overt AAV and its presence is considered an epiphenomenon due to hyperstimulation of the immune system by the infection [143,144,145]. Considering ANCA are pathogenic in AAV, whether ANCA+ COVID-19 patients may develop overt AAV during follow-up is an unanswered question.

Cutaneous vasculitis, a form of SOV, was reported in nine cases. Patients presented with different cutaneous lesions including palpable purpura, hemorrhagic bullae or urticarial lesions [35,36,37,38,39,41,64,65]. Interestingly in five reports, there was a history of empirical antibiotics use for COVID-19 treatment [35,37,41,64,65]. In two contemporary series of cutaneous vasculitis, the main precipitating factors were drugs [146,147]. As such, it is impossible to know if SARS-CoV-2 infection or the antibiotics were the causative factors in those cases.

Regarding large vessel involvement in COVID-19, we found four cases of isolated aortitis and one of GCA. Sollini et al. found an increase in vascular ^18^F-FDG uptake in ^18^F-FDG positron emission tomography/computed tomography (PET/CT) in vascular beds such as the thoracic aorta in recovered adult COVID-19 patients with persisting symptoms [148]. Another study by Dudouet et al. confirmed this phenomenon in the thoracic aorta of 10 out of 47 patients with long COVID; 3 also had abdominal aorta hypermetabolism and 4 out of 5 patients with a follow-up PET/CT scan showed persistent aortic hypermetabolism [149]. It is not known if this phenomenon means active endothelial viral replication, post-infectious aortic inflammation or sequelae [149]. Studies with bigger cohorts and longer follow-ups are needed to answer this question and inform clinicians on how these patients with long COVID must be managed. Dudouet et al. recommend long-term follow-up with vascular imaging to detect morphological changes in the aortic wall of these patients [149].

Other vasculitides reported with less frequency included IgA vasculitis, PAN and anti-GBM disease.

It is important to point out that not all the studies tested for the presence of SARS-CoV-2 directly in the tissue and the possibility that in some cases the vasculitic process could have been provoked by self-limited direct viral-induced vessel damage cannot be ruled out. For example, some cases of large vessel vasculitis resolved without immunosuppressive treatment, which is atypical for GCA or isolated aortitis [44,63].

Finally, regarding vasculitis outcomes, most cases responded well to standard immunosuppressive treatment and had resolved or improved at the last follow-up. Four patients died, but only two were due to the vasculitis manifestations.

Articular involvement was reported during the prodromic phase, the active phase infection or after the recovery of SARS-CoV-2 infection [150,151]. One of the main mechanisms believed to participate in the inflammatory process is molecular mimicry between viral epitopes and the synovial membrane, but other theories considered the presence of circulating immune complexes [152].

In the first scenario (prodromic phase or active infection), cases can be considered as viral-related arthritis. This type of arthritis does not lead to chronic joint disease and was also described in association with hepatitis virus, parvovirus B19, enterovirus, rubella, alphaviruses, flaviviruses, and herpetoviruses [153]. In the present systematic review, none of the authors classified their cases as viral-related arthritis. However, because two patients had arthritis coincident with the SARS-CoV-2 diagnosis, we consider that these patients may fall into this category.

In the second scenario, patients can present articular symptoms after the resolution of SARS-CoV-2 infection. Herein, most of the patients belonged to this group. Furthermore, some of them will fulfill specific classification criteria of rheumatic autoimmune disease, such as RA or SpA. In this systematic review, we found only six patients with RA. In this context, it was hypothesized that these cases correspond to RA triggered by the virus, probably through the production of rheumatoid factor and anti-citrullinated protein antibodies (ACPA). Indeed, flares of RA after SARS-CoV-2 infection were also described [88]. Whether these “induced antibodies” differ from those normally found in RA is unclear. According to Derksen et al., the prevalence of ACPA is not increased after SARS-CoV-2 infection, and RA after COVID-19 may be a coincidence rather than an association [88]. However, we do not know how the clinical course of these patients will be at a longer follow-up. In this regard, all the cases in this systematic review achieved remission at the end of follow-up but were still under immunosuppressors or biologic treatment.

On the other hand, we identified nine patients with SpA. SpA forms a group of overlapping inflammatory rheumatologic diseases involving the joints of the axial skeleton, entheses and peripheral joints. The most common and characteristic initial symptom is chronic low back pain or in the buttock region, usually with an insidious onset [32]. Herein we observed an axial presentation in three patients and a peripheral presentation in six patients. It is important to mention that the ASAS group established the age of 45 years as an eligibility criterion for axial complaints due to the higher prevalence of the disease onset in younger individuals. Based on these criteria, we excluded four patients with axial symptoms and sacroiliitis by MRI. Whether a broader age should be considered for classifying these patients with axial SpA in the context of post-SARS-CoV-2 infection, is still uncertain.

Finally, most of the remaining patients were classified according to the authors as “reactive arthritis”. Traditionally, ReA is defined as sterile arthritis that occurs 1–4 weeks after a genitourinary or gastrointestinal bacterial infection. It is often mono or oligoarticular with an asymmetric distribution and typically affects the lower limbs. While other bacteria and viruses were proposed, they are not included as causative agents of ReA by “classic” definition. For instance, the human immunodeficiency virus (HIV) was observed to cause ReA in some patients [154]. Herein, we only observed balanitis in one of the patients, but no microorganism was isolated. Moreover, not all the patients had a broad microbiological investigation with cultures (blood, urine and stool), urethral swab, or serological tests for bacteria associated with reactive arthritis. Thus probably the term, post-COVID-19 arthritis is more appropriate. Finally, most of the patients in this group responded well to NSAIDs or too short courses of steroids or intra-articular injection, and the symptoms resolved. Nevertheless, due to a short follow-up in these patients, it is still unknown if some of them would have a chronic behavior.

Viral agents that were proposed to be triggers of IIM include Coxsackie B virus, parvovirus, enterovirus, human T-cell lymphotropic virus (HTLV-1), and HIV, through alterations in the cellular host proteins, thereby changing the immune tolerance and via molecular mimicry between pathogens and host proteins that lead to cross-reactivity [155,156]. In patients with SARS-CoV-2 infection, myalgia is one the most prevalent constitutional symptoms reported in the literature. According to a systematic review that included 24,410 patients, myalgia was present in 17% [157]. Muscular injury (defined as the presence of myalgias and CK > 200 U/L) was documented in 10% of hospitalized patients by COVID-19 and some studies have reported increased levels of CK in association with COVID-19 severity [158,159]. The clinical spectrum of muscle damage due to SARS-CoV-2 infection varies from an asymptomatic elevation of CK to severe rhabdomyolysis [160]. The pathophysiology of myositis in SARS-CoV-2 infection is not fully understood. The postulated mechanisms include direct viral muscle invasion (because the skeletal muscle cells express angiotensin-converting enzyme 2 (ACE2), being the receptor involved in SARS-CoV-2 uptake to initiate infection.), immune complex deposition in muscles, the release of myotoxic cytokines, damage due to homology between viral antigens and human muscle cells, and adsorption of viral protein on muscle membranes leading to expression of viral antigens on the myocyte surface [161,162]. However, although muscle injury is relatively common, the presence of autoimmune myositis has been rarely reported. In our review, only nine patients fulfilled the idiopathic IIM classification criteria.

Viruses such as the human endogenous retroviruses, Epstein–Barr virus, parvovirus B19, cytomegalovirus, and HIV have long been considered as possible triggers for SLE [163]. The new SARS-CoV-2 was shown to be able to induce the development of diverse autoantibodies including ANA, anti-Ro/SSA, and antiphospholipid antibodies [6,164]. However, only six patients were reported with a diagnosis of new-onset SLE, and, in four cases, the manifestations began during the acute phase of COVID-19, which can be a challenge for the diagnosis because both diseases share clinical and laboratory characteristics. For instance, general symptoms (fever, fatigue, arthralgia), or lymphopenia are common in both COVID-19 and SLE and are therefore not useful in the differential diagnosis between both diseases. However, based on the clinical findings shown in the cases included in this review, the keys that might help in the differential diagnosis are severe thrombocytopenia, serositis, and kidney damage unrelated to the severity of the respiratory disease. In patients with SARS-CoV-2 infection, thrombocytopenia <100,000/μL is unusual (present in only 5% of patients admitted to an intensive care unit), pleural effusion is detected by chest CT in only 11% and kidney injury is associated with the severity of illness (reported in >50% in patients admitted to the intensive care unit) [165,166,167]. Determination of autoantibodies would confirm or rule out SLE.

In sarcoidosis, it was suggested that SARS-CoV-2 might trigger the formation of noncaseating granulomas via the renin–angiotensin system and the innate immune system [123]. Similar to the other autoimmune diseases, longer follow-ups are required to confirm the diagnosis or to differentiate it from transient symptoms due to the host’s immune response to COVID-19.

The type of immune-related manifestations could be linked with the severity of SARS-CoV-2 infection. More than 50% of the cases of articular diseases (RA, SpA and ReA) appeared in patients with mild COVID-19. In contrast, more than 50% of IIM cases occurred in patients with severe and critical COVID-19, while vasculitis cases were uncommon in this COVID-19 subset. In addition, IIM and SLE presented predominantly during the acute phase of SARS-CoV-2 infection, while the rest presented after the acute phase; this suggests an involvement of different pathogenic mechanisms. Moreover, this spectrum of autoimmune manifestations with onset in different stages of COVID-19 along with differentiated temporal distribution could be taken into count for an early diagnosis. Similar to what occurs with autoimmune diseases unrelated to COVID-19, cases of IIM and SLE were reported more frequently in women while SpA and ReA cases occurred in men. On the other hand, cases of vasculitis predominated in males. The clinical behavior of the new-onset rheumatic autoimmune diseases seemed similar to the non-COVID-19-related counterparts, with the exception of adult-onset Kawasaki disease which displayed the differences mentioned before. Concerning the treatment of COVID-19 associated autoimmune diseases, the patients received similar management as their non-COVID-19-related counterparts. This approach seems to be a reasonable option. In relation to prognosis, it is still uncertain whether rheumatic autoimmune diseases following COVID-19 have a different long-term disease course given the limited information regarding their follow-up.

This review has several limitations owing to its retrospective nature and the heterogeneity among the case reports. In addition, COVID-19 is still under study and the full spectrum of autoimmune phenomena that it may cause in patients during or after SARS-CoV-2 infection is still unknown. However, this systematic review can provide meaningful information on the clinical profile, diagnosis, and management of this complex situation.

## 5. Conclusions

In summary, new-onset rheumatic autoimmune diseases in COVID-19 patients are rare. Most of the cases were vasculitis and arthritis; whereas IIM, SLE, sarcoidosis, systemic sclerosis and AOSD were found in a limited number of patients. The identification of clinical and laboratory features of those autoimmune rheumatic diseases during or following COVID-19 will allow a timely diagnosis and treatment of these conditions. Complementary studies are needed to examine the link between SARS-CoV-2 infection and new onset-rheumatic diseases. Longer follow-up of these patients is also needed to clarify the natural course of these infection-triggered autoimmune conditions.

## Figures and Tables

**Table 1:** Summary of main reported cases of new-onset rheumatic autoimmune diseases during or after SARS-CoV-2 infection.

	Small-Vessel Vasculitis (n = 28)	Medium-Vessel Vasculitis (n = 13)	Large-Vessel Vasculitis (n = 5)	RA (n = 6)	SpA (n = 9)	Reactive Arthritis (n = 17)	Inflammatory Myopathies (n = 9)	SLE (n = 6)
Age, mean ± SD, years	49.9 ± 18	37.3 ± 13.5	64.8 ± 7.9	58.8 ± 14.4	44.1 ± 13.5	50 ± 14.1	55.6 ± 11.5	39.2 ± 24.7
Male, n (%)	17 (60.1)	7 (53.8)	4 (80)	3 (50)	6 (66.6)	9 (52.9)	3 (33.3)	2 (33.3)
Geographical distribution								
Europe, n (%)	10 (35.7)	6 (46.2)	3 (60)	6 (100)	6 (66.6)	9 (52.9)	4 (44.4)	1 (16.7)
Asia, n (%)	8 (28.6)	1 (7.7)	1 (20)	0	2 (22.2)	6 (35.2)	2 (22.2)	1 (16.7)
North America, n (%)	10 (35.7)	6 (46.2)	1 (29)	0	1 (11.1)	2 (11.7)	3 (33.3)	2 (33.3)
South America, n (%)	0	0	0	0	0	0	0	0
Other, n (%)	0	0	0	0	0	0	0	2 (33.3)
COVID-19 severity								
Asymptomatic, n (%)	2 (8)	1 (7.7)	1 (20)	0	0	0		0
Mild, n (%)	6 (24)	7 (53.8)	2 (40)	3 (50)	7 (77.7)	11 (64.7)	1 (11.1)	0
Moderate, n (%)	10 (40)	4 (30.8)	2 (40)	1 (16)	1 (11.1)	1 (5,8)	1 (11.1)	2 (33.3)
Severe, n (%)	4 (16)	1 (7.7)	0	2 (33.3)	1 (11.1)	4 (23.5)	4 (44.4)	0
Critical, n (%)	3 (12)	0	0	0	0	1 (5.8)	1 (11.1)	2 (33.3)
COVID-19 diagnostic method								
Nasopharyngeal swab (RT-PCR or other method) n (%)	21 (77.8)	10 (76.9)	2 (40)	2 (66)	8 (88.8)	16 (94.1)	8 (88.8)	4 (66.7)
Serology, n (%)	3 (11.1)	3 (23.1)	3 (60)	1 (33.3)	3 (33.3)	0	1 (11.1)	2 (33.3)
Imaging, n (%)	2 (7.4)	0	0	0	0	0		0
Epidemiological background, n (%)	1 (3.7)	0	0	0	0	0		0
RAD onset at acute phase of COVID-19, n (%)	11 (44)	7/12	3 (60)	2 (33.3)	0	1 (5.8)	6 (66.6)	4 (66.7)
RAD onset after the acute phase of COVID-19, n (%)	14 (56)	5 (38.5)	2 (40)	4 (66.6)	9 (100)	16 (94.1)	3 (33.3)	2 (33.3)
Days from COVID-19 to ARD onset, mean ± SD, days	28.2 ± 17.7	29.8 ± 8.2	44 ± 16	25.6 ± 12.6	20.7 ± 6.6	19 ± 11.4	30.1 ± 27	24.5 ± 25
COVID-19 outcome								
Resolved, n (%)	23 (92)	13 (100)	5 (100)	3 (50)	9 (100)	16 (94.1)	8 (88.8)	4 (66.7)
Death, n (%)	2 (8)	0	0	0	0	1 (5.8)	1 (11.1)	2 (33.3)
RAD outcome								
Improved or resolved *, n (%)	21 (87.5)	12 (100)	4 (100)	3 (100)	7 (77.7)	16 (94.1)	8 (88.8))	4 (66.7)
Death, n (%)	3 (12.5)	0	0	0	0	1 (6.2)	1 (11.1)	2 (33.3)

* Improved or resolved = defined by the significant improvement of clinical symptoms Abbreviations: COVID-19, coronavirus disease 2019; RA, rheumatoid arthritis; RAD, rheumatic autoimmune diseases; RT-PCR, real-time polymerase chain reaction; SD, standard deviation; SLE, systemic lupus erythematosus; SpA.

## Supplementary Materials

The following are available online at https://www.mdpi.com/article/10.3390/cells10123592/s1, Supplementary file: Reported cases of adult patients with new-onset rheumatic autoimmune diseases during or after COVID-19.

## References

[1] Winchester N.E., Calabrese C., Calabrese L. (2021). The intersection of COVID-19 and autoimmunity: What is our current understanding?. Pathog. Immun..

[2] Smatti M.K., Cyprian F.S., Nasrallah G.K., Al Thani A.A., Almishal R.O., Yassine H.M. (2019). Viruses and Autoimmunity: A Review on the Potential Interaction and Molecular Mechanisms. Viruses.

[3] Hussein H.M., Rahal E.A. (2019). The role of viral infections in the development of autoimmune diseases. Crit. Rev. Microbiol..

[4] Getts D.R., Chastain E.M.L., Terry R.L., Miller S.D. (2013). Virus infection, antiviral immunity, and autoimmunity. Immunol. Rev..

[5] Münz C., Lünemann J.D., Getts M.T., Miller S.D. (2009). Antiviral immune responses: Triggers of or triggered by autoimmunity?. Nat. Rev. Immunol..

[6] Gazzaruso C., Stella N.C., Mariani G., Nai C., Coppola A., Naldani D., Gallotti P. (2020). High prevalence of antinuclear antibodies and lupus anticoagulant in patients hospitalized for SARS-CoV2 pneumonia. Clin. Rheumatol..

[7] Zhou Y., Han T., Chen J., Hou C., Hua L., He S., Guo Y., Zhang S., Wang Y., Yuan J. (2020). Clinical and Autoimmune Characteristics of Severe and Critical Cases of COVID-19. Clin. Transl. Sci..

[8] Woodruff M.C., Ramonell R.P., Saini A.S., Haddad N.S., Anam F.A., Rudolph M.E., Bugrovsky R., Hom J., Cashman K.S., Nguyen D.C. (2021). Relaxed Peripheral Tolerance Drives Broad de Novo Autoreactivity in Severe COVID-19. medRxiv.

[9] Bastard P., Rosen L.B., Zhang Q., Michailidis E., Hoffmann H.-H., Zhang Y., Dorgham K., Philippot Q., Rosain J., Béziat V. (2020). Auto-antibodies against type I IFNs in patients with life-threatening COVID-19. Science.

[10] Mohkhedkar M., Venigalla S.S.K., Janakiraman V. (2021). Untangling COVID-19 and autoimmunity: Identification of plausible targets suggests multi organ involvement. Mol. Immunol..

[11] Shah S., Danda D., Kavadichanda C., Das S., Adarsh M.B., Negi V.S. (2020). Autoimmune and rheumatic musculoskeletal diseases as a consequence of SARS-CoV-2 infection and its treatment. Rheumatol. Int..

[12] Zacharias H., Dubey S., Koduri G., D’Cruz D. (2021). Rheumatological complications of Covid-19. Autoimmun. Rev..

[13] Ramos-Casals M., Brito-Zerón P., Mariette X. (2021). Systemic and organ-specific immune-related manifestations of COVID-19. Nat. Rev. Rheumatol..

[14] Page M.J., McKenzie J.E., Bossuyt P.M., Boutron I., Hoffmann T.C., Mulrow C.D., Shamseer L., Tetzlaff J.M., Akl E.A., Brennan S.E. (2021). The PRISMA 2020 statement: An updated guideline for reporting systematic reviews. BMJ.

[15] Aletaha D., Neogi T., Silman A.J., Funovits J., Felson D., Bingham C.O., Birnbaum N.S., Burmester G.R., Bykerk V.P., Cohen M.D. (2010). 2010 Rheumatoid arthritis classification criteria: An American College of Rheumatology/European League Against Rheumatism collaborative initiative. Arthritis Rheum..

[16] Sieper J., Braun J., Kingsley G.H. (2000). Report on the fourth International workshop on reactive arthritis. Arthritis Rheum..

[17] Jennette J.C., Falk R.J., Bacon P.A., Basu N., Cid M.C., Ferrario F., Flores-Suarez L.F., Gross W.L., Guillevin L., Hagen E.C. (2013). 2012 Revised International Chapel Hill Consensus Conference Nomenclature of Vasculitides. Arthritis Rheum..

[18] Hunder G.G., Bloch D.A., Michel B.A., Stevens M.B., Arend W.P., Do L.H.C., Edworthy S.M., Fauci A.S., Leavitt R.Y., Lie J.T. (1990). The American College of Rheumatology 1990 criteria for the classification of giant cell arteritis. Arthritis Rheum..

[19] Lightfoot R.W., Michel B.A., Bloch D.A., Hunder G.G., Zvaifler N.J., McShane D.J., Arend W.P., Do L.H.C., Leavitt R.Y., Lie J.T. (1990). The American college of rheumatology 1990 criteria for the classification of polyarteritis nodosa. Arthritis Rheum..

[20] Watts R., Lane S., Hanslik T., Hauser T., Hellmich B., Koldingsnes W., Mahr A., Segelmark M., Cohen-Tervaert J.W., Scott D. (2006). Development and validation of a consensus methodology for the classification of the ANCA-associated vasculitides and polyarteritis nodosa for epidemiological studies. Ann. Rheum. Dis..

[21] Kawasaki Disease Case Definition. https://www.cdc.gov/kawasaki/case-definition.html.

[22] McCrindle B.W., Rowley A.H., Newburger J.W., Burns J.C., Bolger A.F., Gewitz M., Baker A.L., Jackson M.A., Takahashi M., Shah P.B. (2017). Diagnosis, Treatment, and Long-Term Management of Kawasaki Disease: A Scientific Statement for Health Professionals From the American Heart Association. Circulation.

[23] Kanegaye J.T., Wilder M.S., Molkara D., Frazer J.R., Pancheri J., Tremoulet A.H., Watson V.E., Best B.M., Burns J.C. (2009). Recognition of a Kawasaki Disease Shock Syndrome. Pediatrics.

[24] Multisystem Inflammatory Syndrome in Adults (MIS-A) Case Definition. https://www.cdc.gov/mis/mis-a/hcp.html.

[25] Multisystem Inflammatory Syndrome in Children (MIS-C) Case Definition. https://www.cdc.gov/mis/mis-c.html.

[26] Bohan A., Peter J.B. (1975). Polymyositis and Dermatomyositis (First of Two Parts). N. Engl. J. Med..

[27] Bohan A., Peter J.B. (1975). Polymyositis and Dermatomyositis (Second of Two Parts). N. Engl. J. Med..

[28] Lundberg E.I., Tjärnlund A., Bottai M., Werth V.P., Pilkington C., De Visser M., Alfredsson L., Amato A., Barohn R.J., Liang M.H. (2017). 2017 European League Against Rheumatism/American College of Rheumatology classification criteria for adult and juvenile idiopathic inflammatory myopathies and their major subgroups. Ann. Rheum. Dis..

[29] Aringer M., Costenbader K., Daikh D., Brinks R., Mosca M., Ramsey-Goldman R., Smolen J.S., Wofsy D., Boumpas D.T., Kamen D.L. (2019). 2019 European League Against Rheumatism/American College of Rheumatology Classification Criteria for Systemic Lupus Erythematosus. Arthritis Rheumatol..

[30] Shiboski C.H., Shiboski S.C., Seror R., Criswell L.A., Labetoulle M., Lietman T.M., Rasmussen A., Scofield H., Vitali C., Bowman S.J. (2017). 2016 American College of Rheumatology/European League Against Rheumatism classification criteria for primary Sjögren’s syndrome: A Consensus and Data-Driven Methodology Involving Three International Patient Cohorts. Ann. Rheum. Dis..

[31] Yamaguchi M., Ohta A., Tsunematsu T., Kasukawa R., Mizushima Y., Kashiwagi H., Kashiwazaki S., Tanimoto K., Matsumoto Y., Ota T. (1992). Preliminary criteria for classification of adult Still’s disease. J. Rheumatol..

[32] Rudwaleit M., Van Der Heijde D., Landewé R., Listing J., Akkoç N., Brandt J., Braun J., Chou C.T., Estévez E.C., Dougados M. (2009). The development of Assessment of SpondyloArthritis international Society classification criteria for axial spondyloarthritis (part II): Validation and final selection. Ann. Rheum. Dis..

[33] (1999). Statement on Sarcoidosis. Am. J. Respir. Crit. Care Med..

[34] Miyakis S., Lockshin M.D., Atsumi T., Branch D.W., Brey R.L., Cervera R., Derksen R.H.W.M., De Groot P.G., Koike T., Meroni P.L. (2006). International consensus statement on an update of the classification criteria for definite antiphospholipid syndrome (APS). J. Thromb. Haemost..

[35] Caputo V., Schroeder J., Rongioletti F. (2020). A generalized purpuric eruption with histopathologic features of leucocytoclastic vasculitis in a patient severely ill with COVID-19. J. Eur. Acad. Dermatol. Venereol..

[36] Mayor-Ibarguren A., Feito-Rodriguez M., Castanedo L.Q., Ruiz-Bravo E., Vega D.M., Herranz-Pinto P. (2020). Cutaneous small vessel vasculitis secondary to COVID-19 infection: A case report. J. Eur. Acad. Dermatol. Venereol..

[37] Iraji F., Galehdari H., Siadat A.H., Jazi S.B. (2021). Cutaneous leukocytoclastic vasculitis secondary to COVID-19 infection: A case report. Clin. Case Rep..

[38] Dominguez-Santas M., Diaz-Guimaraens B., Abellas P.G., Del Real C.M., Burgos-Blasco P., Suarez-Valle A. (2020). Cutaneous small-vessel vasculitis associated with novel 2019 coronavirus SARS-CoV-2 infection (COVID-19). J. Eur. Acad. Dermatol. Venereol..

[39] Kösters K., Schwarzer S., Labuhn A., Rübben A., Yang S., Hessler F., Assaf C. (2020). Cutaneous Vasculitis in a Patient With COVID-19. Open Forum Infect. Dis..

[40] Shaigany S., Gnirke M., Guttmann A., Chong H., Meehan S., Raabe V., Louie E., Solitar B., Femia A. (2020). An adult with Kawasaki-like multisystem inflammatory syndrome associated with COVID-19. Lancet.

[41] Negrini S., Guadagno A., Greco M., Parodi A., Burlando M. (2020). An unusual case of bullous haemorrhagic vasculitis in a COVID-19 patient. J. Eur. Acad. Dermatol. Venereol..

[42] Duran T.I., Turkmen E., Dilek M., Sayarlioglu H., Arik N. (2021). ANCA-associated vasculitis after COVID-19. Rheumatol. Int..

[43] Hussein A., Al Khalil K., Bawazir Y.M. (2020). Anti-Neutrophilic Cytoplasmic Antibody (ANCA) Vasculitis Presented as Pulmonary Hemorrhage in a Positive COVID-19 Patient: A Case Report. Cureus.

[44] Oda R., Inagaki T., Ishikane M., Hotta M., Shimomura A., Sato M., Nakamoto T., Akiyama Y., Yamamoto K., Minamimoto R. (2020). Case of adult large vessel vasculitis after SARS-CoV-2 infection. Ann. Rheum. Dis..

[45] Timmons G.M., Rempe T., Bevins E.A., Goodwill V., Miner A., Kavanaugh A., Ritter M., Graves J.S. (2021). CNS Lymphocytic Vasculitis in a Young Woman With COVID-19 Infection. Neurol.-Neuroimmunol. Neuroinflamm..

[46] Chérif M.Y., de Filette J.M., André S., Kamgang P., Richert B., Clevenbergh P. (2020). Coronavirus disease 2019–related Kawasaki-like disease in an adult: A case report. JAAD Case Rep..

[47] Sokolovsky S., Soni P., Hoffman T., Kahn P., Scheers-Masters J. (2021). COVID-19 associated Kawasaki-like multisystem inflammatory disease in an adult. Am. J. Emerg. Med..

[48] Vacchi C., Meschiari M., Milic J., Marietta M., Tonelli R., Alfano G., Volpi S., Faltoni M., Franceschi G., Ciusa G. (2020). COVID-19-associated vasculitis and thrombotic complications: From pathological findings to multidisciplinary discussion. Rheumatology.

[49] Selvaraj V., Moustafa A., Dapaah-Afriyie K., Birkenbach M.P. (2021). COVID-19-induced granulomatosis with polyangiitis. BMJ Case Rep..

[50] Allez M., Denis B., Bouaziz J., Battistella M., Zagdanski A., Bayart J., Lazaridou I., Gatey C., Pillebout E., Baudier M.C. (2020). COVID-19–Related IgA Vasculitis. Arthritis Rheumatol..

[51] Li N.L., Papini A.B., Shao T., Girard L. (2021). Immunoglobulin-A Vasculitis With Renal Involvement in a Patient With COVID-19: A Case Report and Review of Acute Kidney Injury Related to SARS-CoV-19. Can. J. Kidney Health Dis..

[52] Kudose S., Batal I., Santoriello D., Xu K., Barasch J., Peleg Y., Canetta P., Ratner L.E., Marasa M., Gharavi A.G. (2020). Kidney Biopsy Findings in Patients with COVID-19. J. Am. Soc. Nephrol..

[53] Uppal N.N., Kello N., Shah H.H., Khanin Y., De Oleo I.R., Epstein E., Sharma P., Larsen C.P., Bijol V., Jhaveri K.D. (2020). De Novo ANCA-Associated Vasculitis With Glomerulonephritis in COVID. Kidney Int. Rep..

[54] Lind E., Jameson A., Kurban E. (2021). Fulminant granulomatosis with polyangiitis presenting with diffuse alveolar haemorrhage following COVID. BMJ Case Rep..

[55] Barbetta L., Filocamo G., Passoni E., Boggio F., Folli C., Monzani V. (2021). Henoch-Schönlein Purpura with Renal and Gastro-intestinal Involvement in Course of COVID-19: A Case Report. Clin. Exp. Rheumatol..

[56] Suso A.S., Mon C., Alonso I.O., Romo K.G., Juarez R.C., Ramírez C.L., Sánchez M.S., Valdivia V.M., Librero M.O., Pala A.O. (2020). IgA Vasculitis With Nephritis (Henoch−Schönlein Purpura) in a COVID-19 Patient. Kidney Int. Rep..

[57] Patel P., DeCuir J., Abrams J., Campbell A.P., Godfred-Cato S., Belay E.D. (2021). Clinical Characteristics of Multisystem Inflammatory Syndrome in Adults. JAMA Netw. Open.

[58] Dixon L., Coughlan C., Karunaratne K., Gorgoraptis N., Varley J., Husselbee J., Mallon D., Carroll R., Jones B., Boynton C. (2021). Immunosuppression for intracranial vasculitis associated with SARS-CoV-2: Therapeutic implications for COVID-19 cerebrovascular pathology. J. Neurol. Neurosurg. Psychiatry.

[59] Cogan E., Foulon P., Cappeliez O., Dolle N., Vanfraechem G., De Backer D. (2020). Multisystem Inflammatory Syndrome With Complete Kawasaki Disease Features Associated With SARS-CoV-2 Infection in a Young Adult. A Case Report. Front. Med..

[60] Bressler M.Y., Pathak N., Cervellione K., Bagheri F., Epstein E., Mir A., Tamez R. (2021). New Onset Granulomatosis with Polyangiitis Associated with COVID-19. Case Rep. Dermatol. Med..

[61] Moeinzadeh F., Dezfouli M., Naimi A., Shahidi S., Moradi H. (2020). Newly Diagnosed Glomerulonephritis During COVID-19 Infection Undergoing Immunosuppression Therapy, a Case Report. Iran J. Kidney Dis..

[62] Sandhu S., Chand S., Bhatnagar A., Dabas R., Bhat S., Kumar H., Dixit P.K. (2021). Possible association between IgA vasculitis and COVID-19. Dermatol. Ther..

[63] Riera-Martí N., Romaní J., Calvet J. (2021). SARS-CoV-2 infection triggering a giant cell arteritis. Med. Clínica.

[64] Nasiri S., Dadkhahfar S., Abasifar H., Mortazavi N., Gheisari M. (2020). Urticarial vasculitis in a COVID-19 recovered patient. Int. J. Dermatol..

[65] De Perosanz-Lobo D., Fernandez-Nieto D., Burgos-Blasco P., Selda-Enriquez G., Carretero I., Moreno C., Fernández-Guarino M. (2020). Urticarial vasculitis in COVID-19 infection: A vasculopathy-related symptom?. J. Eur. Acad. Dermatol. Venereol..

[66] Dhakal P., Khadka S., Clowes J.A., Chakinala R.C. (2021). Aortitis in COVID-19. IDCases.

[67] Shergill S., Davies J., Bloomfield J. (2020). Florid aortitis following SARS-CoV-2 infection. Eur. Heart J..

[68] Kuriyama Y., Shimizu A., Oka H., Sato M., Makioka K., Ikota H., Yanagisawa K., Tokue Y., Tsukagoshi H., Motegi S. (2021). Erythema nodosum-like eruption in coronavirus disease 2019: A case report and literature review of Asian countries. J. Dermatol..

[69] Lidder A.K., Pandit S.A., Lazzaro D.R. (2020). An adult with COVID-19 kawasaki-like syndrome and ocular manifestations. Am. J. Ophthalmol. Case Rep..

[70] Malangu B., Quintero J.A., Capitle E.M. (2020). Adult Inflammatory Multi-System Syndrome Mimicking Kawasaki Disease in a Patient With COVID-19. Cureus.

[71] Vergnano S., Alders N., Armstrong C., Bamber A.R., Bandi S., Evans A.J., Hajiani N., Kenny J., Kucera F., Tometzki A. (2020). Severe refractory Kawasaki disease in seven infants in the COVID-19 era. Lancet Rheumatol..

[72] Kofman A.D., Sizemore E.K., Detelich J.F., Albrecht B., Piantadosi A.L. (2020). A young adult with COVID-19 and multisystem inflammatory syndrome in children (MIS-C)-like illness: A case report. BMC Infect. Dis..

[73] Ventura M.J., Guajardo E., Clark E.H., Bhairavarasu K., Kherallah R.Y., Dinardo A.R., Ye X., Piedra A.P., Atmar R.L., Agarwal S.K. (2020). Correspondence on ‘Paediatric multisystem inflammatory syndrome temporally associated with SARS-CoV-2 mimicking Kawasaki disease (Kawa-COVID-19): A multicentre cohort’ by Pouletty et al. Ann. Rheum. Dis..

[74] Jones I., Bell L.C.K., Manson J.J., Last A. (2020). An adult presentation consistent with PIMS-TS. Lancet Rheumatol..

[75] Parker A., Louw E.H., Lalla U., Koegelenberg C.F.N., Allwood B.W., Rabie H., Sibeko I.S., Taljaard J.J., Lahri S. (2020). Multisystem inflammatory syndrome in adult COVID-19 patients. S. Afr. Med. J..

[76] Nagra D., Russell M.D., Rosmini S., Sado D., Buazon A., Shafi T., Hamlyn E., Sandhu G., Rutherford I.A., Galloway J.B. (2021). A Kawasaki-like illness in an adult with recent SARS-CoV-2 infection. Rheumatol. Adv. Pract..

[77] Lechien J.R., Hervochon R., Hans S. (2021). Post-COVID-19 Kawasaki-Like Syndrome. Ear Nose Throat J..

[78] Novelli L., Motta F., Ceribelli A., Guidelli G.M., Luciano N., Isailovic N., Vecellio M., Caprioli M., Clementi N., Clementi M. (2021). A case of psoriatic arthritis triggered by SARS-CoV-2 infection. Rheumatology.

[79] Liew I.Y., Mak T.M., Cui L., Vasoo S., Lim X.R. (2020). A Case of Reactive Arthritis Secondary to Coronavirus Disease 2019 Infection. JCR: J. Clin. Rheumatol..

[80] Kuschner Z., Do A.O., Mukherji P. (2021). A case of SARS-CoV-2-associated arthritis with detection of viral RNA in synovial fluid. J. Am. Coll. Emerg. Phys. Open.

[81] Cincinelli G., Di Taranto R., Orsini F., Rindone A., Murgo A., Caporali R. (2021). A case report of monoarthritis in a COVID-19 patient and literature review. Medicine.

[82] Baimukhamedov C., Barskova T., Matucci-Cerinic M. (2021). Arthritis after SARS-CoV-2 infection. Lancet Rheumatol..

[83] El Hasbani G., Jawad A., Uthman I. (2021). Axial and peripheral spondyloarthritis triggered by sars-cov-2 infection: A report of two cases. Reumatismo.

[84] Di Carlo M., Tardella M., Salaffi F. (2021). Can SARS-CoV-2 Induce Reactive Arthritis?. Clin. Exp. Rheumatol..

[85] Fragata I., Mourão A.F. (2021). Coronavirus Disease 19 (COVID-19) complicated with post-viral arthritis. Acta Reum. Port.

[86] Roongta R., Chattopadhyay A., Ghosh A. (2021). Correspondence on ‘Onset of rheumatoid arthritis after COVID-19: Coincidence or connected?’. Ann. Rheum. Dis..

[87] Derksen V.F.A.M., Kissel T., Lamers-Karnebeek F.B.G., van der Bijl E.A., Venhuizen A.C., Huizinga T.W.J., Toes R.E.M., Roukens E.A.H., van der Woude D. (2021). Onset of rheumatoid arthritis after COVID-19: Coincidence or connected?. Ann. Rheum. Dis..

[88] Gasparotto M., Framba V., Piovella C., Doria A., Iaccarino L. (2021). Post-COVID-19 arthritis: A case report and literature review. Clin. Rheumatol..

[89] Hønge B.L., Hermansen M.-L.F., Storgaard M. (2021). Reactive arthritis after COVID-19. BMJ Case Rep..

[90] Kocyigit B.F., Akyol A. (2021). Reactive arthritis after COVID-19: A case-based review. Rheumatol. Int..

[91] Danssaert Z., Raum G., Hemtasilpa S. (2020). Reactive Arthritis in a 37-Year-Old Female With SARS-CoV2 Infection. Cureus.

[92] Ono K., Kishimoto M., Shimasaki T., Uchida H., Kurai D., Deshpande A.G., Komagata Y., Kaname S. (2020). Reactive arthritis after COVID-19 infection. RMD Open.

[93] Sureja N.P., Nandamuri D. (2021). Reactive arthritis after SARS-CoV-2 infection. Rheumatol. Adv. Pract..

[94] Schenker H.M., Hagen M., Simon D., Schett G., Manger B. (2021). Reactive arthritis and cutaneous vasculitis after SARS-CoV-2 infection. Rheumatology.

[95] Salvatierra J., Martínez-Peñalver D., Salvatierra-Velasco L. (2020). CoVid-19 related dactyitis. Jt. Bone Spine.

[96] Jali I. (2020). Reactive Arthritis After COVID-19 Infection. Cureus.

[97] Talarico R., Stagnaro C., Ferro F., Carli L., Mosca M. (2020). Symmetric peripheral polyarthritis developed during SARS-CoV-2 infection. Lancet Rheumatol..

[98] Saikali W., Gharib S. (2021). The first non-radiographic axial spondyloarthrits with COVID-19. Immunity Inflamm. Dis..

[99] De Stefano L., Rossi S., Montecucco C., Bugatti S. (2020). Transient monoarthritis and psoriatic skin lesions following COVID-19. Ann. Rheum. Dis..

[100] Parisi S., Borrelli R., Bianchi S., Fusaro E. (2020). Viral arthritis and COVID-19. Lancet Rheumatol..

[101] Alivernini S., Cingolani A., Gessi M., Paglionico A., Pasciuto G., Tolusso B., Fantoni M., Gremese E. (2021). Comparative analysis of synovial inflammation after SARS-CoV-2 infection. Ann. Rheum. Dis..

[102] Mukarram M.S. (2020). Jinnah Medical College Hospital Post COVID-19 Reactive Arthritis: An Emerging Existence In The Spectrum Of Musculoskeletal Complications Of SARS-CoV-2 Infection. Clin. Stud. Med Case Rep..

[103] Yokogawa N., Minematsu N., Katano H., Suzuki T. (2021). Case of acute arthritis following SARS-CoV-2 infection. Ann. Rheum. Dis..

[104] Perrot L., Hemon M., Busnel J.-M., Muis-Pistor O., Picard C., Zandotti C., Pham T., Roudier J., Desplat-Jego S., Balandraud N. (2021). First flare of ACPA-positive rheumatoid arthritis after SARS-CoV-2 infection. Lancet Rheumatol..

[105] Gibson M., Sampat K., Coakley G. (2020). EP15 A self-limiting symmetrical polyarthritis following COVID-19 infection. Rheumatol. Adv. Pr..

[106] Uslu S. (2021). Myositis due to COVID-19. Postgrad. Med. J..

[107] Beydon M., Chevalier K., Al Tabaa O., Hamroun S., Delettre A.-S., Thomas M., Herrou J., Riviere E., Mariette X. (2021). Myositis as a manifestation of SARS-CoV-19. Ann. Rheum. Dis..

[108] Shabbir A., Camm C.F., Elkington A., Tilling L., Stirrup J., Chan A., Bull S. (2020). Myopericarditis and myositis in a patient with COVID-19: A case report. Eur. Hearth J.Case Rep..

[109] Lokineni S., Mortezavi M. (2021). Delayed-onset Necrotizing Myositis following COVID-19 Infection. Eur. J. Case Rep. Intern. Med..

[110] Zhang H., Charmchi Z., Seidman R.J., Anziska Y., Do V.V., Perk J. (2020). COVID -19–associated myositis with severe proximal and bulbar weakness. Muscle Nerve.

[111] Veyseh M., Koyoda S., Ayesha B. (2021). COVID-19 IgG-related autoimmune inflammatory necrotizing myositis. BMJ Case Rep..

[112] Sacchi M.C., Tamiazzo S., Lauritano E.C., Bonometti R. (2020). Case Report of COVID-19 in an Elderly Patient: Could SARS-CoV2 Trigger Myositis?. Eur. Rev. Med. Pharmacol. Sci..

[113] Gokhale Y., Patankar A., Holla U., Shilke M., Kalekar L., Karnik N.D., Bidichandani K., Baveja S., Joshi A. (2020). Dermatomy-ositis during COVID-19 Pandemic (A Case Series): Is There a Cause Effect Relationship?. J. Assoc. Physicians. India.

[114] Hali F., Jabri H., Chiheb S., Hafiani Y., Nsiri A. (2021). A concomitant diagnosis of COVID-19 infection and systemic lupus erythematosus complicated by a macrophage activation syndrome: A new case report. Int. J. Dermatol..

[115] Gracia-Ramos A.E., Saavedra-Salinas M. (2021). Ángel Can the SARS-CoV-2 infection trigger systemic lupus erythematosus? A case-based review. Rheumatol. Int..

[116] Cardoso E.M., Hundal J., Feterman D., Magaldi J. (2020). Concomitant new diagnosis of systemic lupus erythematosus and COVID-19 with possible antiphospholipid syndrome. Just a coincidence? A case report and review of intertwining pathophysiology. Clin. Rheumatol..

[117] Slimani Y., Abbassi R., El Fatoiki F., Barrou L., Chiheb S. (2021). Systemic lupus erythematosus and varicella-like rash following COVID-19 in a previously healthy patient. J. Med. Virol..

[118] Zamani B., Taba S.-M.M., Shayestehpour M. (2021). Systemic lupus erythematosus manifestation following COVID-19: A case report. J. Med. Case Rep..

[119] Bonometti R., Sacchi M.C., Stobbione P., Lauritano E.C., Tamiazzo S., Marchegiani A., Novara E., Molinaro E., Benedetti I., Massone L. (2020). The first case of systemic lupus erythematosus (SLE) triggered by COVID-19 infection. Eur. Rev. Med Pharmacol. Sci..

[120] Fineschi S. (2021). Case Report: Systemic Sclerosis After Covid-19 Infection. Front. Immunol..

[121] Bamidis A.D., Koehler P., di Cristanziano V., Rasche K., Demirel B., Bacher P., Hallek M., Kochanek M., Klein F., Hofmann S.C. (2021). First manifestation of adult-onset Still’s disease after COVID-19. Lancet Rheumatol..

[122] Ekinci A.P., Büyükbabani N., Meşe S., Pehlivan G., Okumuş N., Ağaçfidan A., Özkaya E. (2021). COVID-19-triggered sarcoidal granulomas mimicking scar sarcoidosis. J. Eur. Acad. Dermatol. Venereol..

[123] Mertz P., Jeannel J., Guffroy A., Lescuyer S., Korganow A.S., Rondeau-Lutz M., Weber J.C. (2021). Granulomatous manifestations associated with COVID19 infection: Is there a link between these two diseases?. Autoimmun. Rev..

[124] Behbahani S., Baltz J.O., Droms R., Deng A.C., Amano S.U., Levin N.A., O’Brien M.C., Wiss K. (2020). Sarcoid-like reaction in a patient recovering from coronavirus disease 19 pneumonia. JAAD Case Rep..

[125] Colmenero I., Santonja C., Alonso-Riaño M., Noguera-Morel L., Hernández-Martín A., Andina D., Wiesner T., Rodríguez-Peralto J., Requena L., Torrelo A. (2020). SARS-CoV-2 endothelial infection causes COVID-19 chilblains: Histopathological, immunohistochemical and ultrastructural study of seven paediatric cases. Br. J. Dermatol..

[126] McGonagle D., Bridgewood C., Ramanan A.V., Meaney J.F.M., Watad A. (2021). COVID-19 vasculitis and novel vasculitis mimics. Lancet Rheumatol..

[127] Verdoni L., Mazza A., Gervasoni A., Martelli L., Ruggeri M., Ciuffreda M., Bonanomi E., D’Antiga L. (2020). An outbreak of severe Kawasaki-like disease at the Italian epicentre of the SARS-CoV-2 epidemic: An observational cohort study. Lancet.

[128] Pouletty M., Borocco C., Ouldali N., Caseris M., Basmaci R., Lachaume N., Bensaid P., Pichard S., Kouider H., Morelle G. (2020). Paediatric multisystem inflammatory syndrome temporally associated with SARS-CoV-2 mimicking Kawasaki disease (Kawa-COVID-19): A multicentre cohort. Ann. Rheum. Dis..

[129] Feldstein L.R., Rose E.B., Horwitz S.M., Collins J.P., Newhams M.M., Son M.B.F., Newburger J.W., Kleinman L.C., Heidemann S.M., Martin A.A. (2020). Multisystem Inflammatory Syndrome in U.S. Children and Adolescents. N. Engl. J. Med..

[130] Roncati L., Ligabue G., Fabbiani L., Malagoli C., Gallo G., Lusenti B., Nasillo V., Manenti A., Maiorana A. (2020). Type 3 hypersensitivity in COVID-19 vasculitis. Clin. Immunol..

[131] Fraison J.-B., Sève P., Dauphin C., Mahr A., Gomard-Mennesson E., Varron L., Pugnet G., Landron C., Roblot P., Oziol E. (2016). Kawasaki disease in adults: Observations in France and literature review. Autoimmun. Rev..

[132] Chang L.-Y., Lu C.-Y., Shao P.-L., Lee P.-I., Lin M.-T., Fan T.-Y., Cheng A.-L., Lee W.-L., Hu J.-J., Yeh S.-J. (2014). Viral infections associated with Kawasaki disease. J. Formos. Med. Assoc..

[133] Turnier J.L., Anderson M.S., Heizer H.R., Jone P.-N., Glodé M.P., Dominguez S.R. (2015). Concurrent Respiratory Viruses and Kawasaki Disease. Pediatrics.

[134] Guleria S., Jindal A.K., Pandiarajan V., Singh M.P., Singh S. (2018). Dengue-Triggered Kawasaki Disease: A Report of 2 Cases. JCR J. Clin. Rheumatol..

[135] Toubiana J., Cohen J.F., Brice J., Poirault C., Bajolle F., Curtis W., Moulin F., Matczak S., Leruez M., Casanova J.-L. (2021). Distinctive Features of Kawasaki Disease Following SARS-CoV-2 Infection: A Controlled Study in Paris, France. J. Clin. Immunol..

[136] Yener G.O., Kısaarslan A.P., Ulu K., Atalay E., Haşlak F., Özdel S., Yücel B.B., Yıldırım D.G., Çakmak F., Öztürk K. (2021). Differences and similarities of multisystem inflammatory syndrome in children, Kawasaki disease and macrophage activating syndrome due to systemic juvenile idiopathic arthritis: A comparative study. Rheumatol. Int..

[137] Consiglio C.R., Cotugno N., Sardh F., Pou C., Amodio D., Rodriguez L., Tan Z., Zicari S., Ruggiero A., Pascucci G.R. (2020). The Immunology of Multisystem Inflammatory Syndrome in Children with COVID-19. Cell.

[138] Rodriguez-Smith J.J., Verweyen E.L., Clay G.M., Esteban Y.M., de Loizaga S.R., Baker E.J., Do T., Dhakal S., Lang S.M., Grom A.A. (2021). Inflammatory biomarkers in COVID-19-associated multisystem inflammatory syndrome in children, Kawasaki disease, and macrophage activation syndrome: A cohort study. Lancet Rheumatol..

[139] Henderson L.A., Canna S.W., Friedman K.G., Gorelik M., Lapidus S.K., Bassiri H., Behrens E.M., Ferris A., Kernan K.F., Schulert G.S. (2021). American College of Rheumatology Clinical Guidance for Multisystem Inflammatory Syndrome in Children Associated With SARS–CoV-2 and Hyperinflammation in Pediatric COVID-19: Version 2. Arthritis Rheumatol..

[140] Santoriello D., Khairallah P., Bomback A.S., Xu K., Kudose S., Batal I., Barasch J., Radhakrishnan J., D’Agati V., Markowitz G. (2020). Postmortem Kidney Pathology Findings in Patients with COVID-19. J. Am. Soc. Nephrol..

[141] Patel R., Amrutiya V., Baghal M., Shah M., Lo A. (2021). Life-Threatening Diffuse Alveolar Hemorrhage as an Initial Presentation of Microscopic Polyangiitis: COVID-19 as a Likely Culprit. Cureus.

[142] Löffler C., Mahrhold J., Fogarassy P., Beyer M., Hellmich B. (2020). Two Immunocompromised Patients With Diffuse Alveolar Hemorrhage as a Complication of Severe Coronavirus Disease. Chest.

[143] Gelzo M., Cacciapuoti S., Pinchera B., De Rosa A., Cernera G., Scialò F., Comegna M., Mormile M., Gallicchio A., Fabbrocini G. (2021). A Transient Increase in the Serum ANCAs in Patients with SARS-CoV-2 Infection: A Signal of Subclinical Vasculitis or an Epiphenomenon with No Clinical Manifestations? A Pilot Study. Viruses.

[144] Vlachoyiannopoulos P.G., Magira E., Alexopoulos H., Jahaj E., Theophilopoulou K., Kotanidou A., Tzioufas A.G. (2020). Autoantibodies related to systemic autoimmune rheumatic diseases in severely ill patients with COVID-19. Ann. Rheum. Dis..

[145] Dotan A., Muller S., Kanduc D., David P., Halpert G., Shoenfeld Y. (2021). The SARS-CoV-2 as an instrumental trigger of autoimmunity. Autoimmun. Rev..

[146] Pastuszczak M., Celińska-Löwenhoff M., Sułowicz J., Wojas-Pelc A., Musiał J. (2017). Clinical study on single-organ cutaneous small vessels vasculitis (SoCSVV). Medicine.

[147] Loricera J., Blanco R., Ortiz-Sanjuán F., Hernández J.L., Pina T., González-Vela M.C., Calvo-Río V., Rueda-Gotor J., Alvarez L., González-López M.A. (2015). Single-organ cutaneous small-vessel vasculitis according to the 2012 revised International Chapel Hill Consensus Conference Nomenclature of Vasculitides: A study of 60 patients from a series of 766 cutaneous vasculitis cases. Rheumatology.

[148] Sollini M., Ciccarelli M., Cecconi M., Aghemo A., Morelli P., Gelardi F., Chiti A. (2021). Vasculitis changes in COVID-19 survivors with persistent symptoms: An [18F] FDG-PET/CT study. Eur. J. Nucl. Med. Mol. Imaging.

[149] Dudouet P., Cammilleri S., Guedj E., Jacquier A., Raoult D., Eldin C. (2021). Aortic 18F-FDG PET/CT hypermetabolism in patients with long COVID: A retrospective study. Clin. Microbiol. Infect..

[150] Wang D., Hu B., Hu C., Zhu F., Liu X., Zhang J., Wang B., Xiang H., Cheng Z., Xiong Y. (2020). Clinical Characteristics of 138 Hospitalized Patients With 2019 Novel Coronavirus-Infected Pneumonia in Wuhan, China. JAMA.

[151] Joob B., Wiwanitkit V. (2020). Arthralgia as an initial presentation of COVID-19: Observation. Rheumatol. Int..

[152] Fujinami R.S., von Herrath M.G., Christen U., Whitton J.L. (2006). Molecular Mimicry, Bystander Activation, or Viral Persistence: Infections and Autoimmune Disease. Clin. Microbiol. Rev..

[153] Vassilopoulos D., Calabrese L.H. (2008). Virally associated arthritis 2008: Clinical, epidemiologic, and pathophysiologic considerations. Arthritis Res. Ther..

[154] Stavropoulos P., Soura E., Kanelleas A., Katsambas A., Antoniou C. (2014). Reactive Arthritis. J. Eur. Acad. Dermatol. Venereol..

[155] Riebeling-Navarro C., Nava A. (2009). Patogenia de las miopatías inflamatorias idiopáticas. Reumatol. Clín..

[156] Adler B., Christopher-Stine L., Cristopher-Stine L. (2018). Triggers of inflammatory myopathy: Insights into pathogenesis. Discov. Med..

[157] Grant M.C., Geoghegan L., Arbyn M., Mohammed Z., McGuinness L., Clarke E.L., Wade R.G. (2020). The prevalence of symptoms in 24,410 adults infected by the novel coronavirus (SARS-CoV-2; COVID-19): A systematic review and meta-analysis of 148 studies from 9 countries. PLoS ONE.

[158] Berth S.H., Lloyd T.E. (2020). Secondary Causes of Myositis. Curr. Treat. Options Neurol..

[159] Tsivgoulis G., Palaiodimou L., Katsanos A.H., Caso V., Köhrmann M., Molina C., Cordonnier C., Fischer U., Kelly P., Sharma V. (2020). Neurological manifestations and implications of COVID-19 pandemic. Ther. Adv. Neurol. Disord..

[160] Saud A., Naveen R., Aggarwal R., Gupta L. (2021). COVID-19 and Myositis: What We Know So Far. Curr. Rheumatol. Rep..

[161] Gracia-Ramos A.E. (2020). Is the ACE2 Overexpression a Risk Factor for COVID-19 Infection?. Arch. Med. Res..

[162] Paliwal V.K., Garg R.K., Gupta A., Tejan N. (2020). Neuromuscular presentations in patients with COVID-19. Neurol. Sci..

[163] Rigante D., Mazzoni M.B., Esposito S. (2014). The cryptic interplay between systemic lupus erythematosus and infections. Autoimmun. Rev..

[164] Fujii H., Tsuji T., Yuba T., Tanaka S., Suga Y., Matsuyama A., Omura A., Shiotsu S., Takumi C., Ono S. (2020). High levels of anti-SSA/Ro antibodies in COVID-19 patients with severe respiratory failure: A case-based review: High Levels of Anti-SSA/Ro Antibodies in COVID-19. Clin. Rheumatol..

[165] Huang C., Wang Y., Li X., Ren L., Zhao J., Hu Y., Zhang L., Fan G., Xu J., Gu X. (2020). Clinical features of patients infected with 2019 novel coronavirus in Wuhan, China. Lancet.

[166] Sun Z., Zhang N., Li Y., Xu X. (2020). A systematic review of chest imaging findings in COVID-19. Quant. Imaging Med. Surg..

[167] Nadim M.K., Forni L.G., Mehta R.L., Connor M.J., Liu K.D., Ostermann M., Rimmelé T., Zarbock A., Bell S., Bihorac A. (2020). COVID-19-associated acute kidney injury: Consensus report of the 25th Acute Disease Quality Initiative (ADQI) Workgroup. Nat. Rev. Nephrol..

